# Experience of Using Wearable Devices for Dietary Management for Chinese Americans With Type 2 Diabetes: One-Group Prospective Cohort Study

**DOI:** 10.2196/73381

**Published:** 2025-10-02

**Authors:** Michaela Greenlee, Bei Wu, Mingui Sun, Keer Chen, Wenyan Jia, Susan Zweig, Gail Melkus, Niyati Parekh, Yaguang Zheng

**Affiliations:** 1 Harvard T.H. Chan School of Public Health, 677 Huntington Ave, Boston, MA, 02215, United States, 1 7038263278; 2 NYU Shanghai, Shanghai, China; 3 Department of Neurological Surgery, School of Medicine, University of Pittsburgh, Pittsburgh, PA, United States; 4 Department of Nutrition and Food Studies, Steinhardt School of Culture, Education, and Human Development, New York University, New York, NY, United States; 5 Division of Endocrinology, Department of Medicine, NYU Grossman School of Medicine, New York, NY, United States; 6 NYU Rory Meyers College of Nursing, New York, NY, United States; 7 School of Global Public Health, NYU Public Health Nutrition, New York, NY, United States

**Keywords:** Chinese Americans, continuous glucose monitor, dietary management, eButton, type 2 diabetes, wearable devices, electronic button

## Abstract

**Background:**

Chinese Americans with type 2 diabetes (T2D) face significant challenges in dietary management, which is crucial for glycemic control. Wearable sensors, such as the electronic button (eButton) and continuous glucose monitor (CGM), offer a promising solution.

**Objective:**

We aimed to explore the experience of using the eButton and CGM for dietary management among Chinese Americans with T2D.

**Methods:**

Chinese Americans with T2D (N=11) participated in a one-group prospective cohort study, recruited via convenience sampling from the electronic medical records of NYU Langone Health. Participants wore an eButton on their chest to record their 10-day meals and a CGM for the 2 weeks and kept a diary to track food intake, medication, and physical activity. Individual interviews were conducted after 2 weeks to discuss their experience, barriers, and facilitators of use. Interview transcripts were thematically analyzed using ATLAS.ti (Scientific Software Development GmbH) software.

**Results:**

Facilitators of using an eButton included the device’s ease of use, ability to make participants more mindful, and influence on increased sense of control. Greater awareness of food intake enabled participants to eat smaller portions. Reported barriers included privacy concerns, difficulty positioning the camera for pictures, and the lack of a meal photo record to track glucose trends. For the CGM, facilitators included its comfort and ease of use, its ability to increase mindfulness of meal choices, and its motivating changes in eating behaviors. The most common barriers included the sensor falling off, getting trapped in clothes, and causing skin sensitivity.

**Conclusions:**

Our findings suggest that it is feasible for Chinese Americans with T2D to use eButton and CGM for dietary management. When paired, these tools offer a promising method to help patients visualize the relationship between food intake and glycemic response. For clinical implementation, structured support from health care providers—such as dietitians or diabetes educators—is essential to help patients interpret the data meaningfully. Clinicians should also consider cultural factors, privacy concerns, and individual preferences when introducing wearable technologies, ensuring a personalized and patient-centered approach to diabetes care. Future studies should apply these devices to a larger sample over a longer duration to better inform effective diabetes management strategies.

## Introduction

Type 2 diabetes (T2D) is becoming a growing public health concern in the United States and globally due to rapid cultural and social changes, such as rising urbanization, aging populations, and maladaptive lifestyle behaviors (eg, reduced physical activity and poor diet) [1]. This disease is associated with detrimental health outcomes, including stroke, cardiovascular disease, and kidney failure. The Centers for Disease Control reports the age-adjusted prevalence rate of T2D among Asian Americans is 9%, placing them at moderate risk [2]. Notably, the age-adjusted prevalence rate among Chinese Americans increased from 8.69% to 10.57% between 2011 and 2018, likely paralleling rising immigration levels [3]. Other studies have found that Chinese Americans are also at a higher risk for T2D than other racial and ethnic groups despite having a lower average BMI [4]. Chinese Americans’ higher risk of developing T2D may be partly explained by eating certain cultural staple foods that typically elicit a high glycemic response, including rice, noodles, and steamed buns [5]. Moreover, unhealthy eating habits may be exacerbated by acculturation, as exposure to Western dietary patterns often includes foods with higher levels of fat, sugar, and sodium.

Diabetes dietary self-management is a critical part of managing the disease to achieve optimal glycemic control. The dietary recommendation guidelines proposed by the American Diabetes Association (ADA) [6,7] suggest that the daily intake of carbohydrates be less than 130 grams and that patients should avoid consuming food rich in starch. However, Chinese Americans’ ability to practice these dietary management recommendations is challenging. It has been found that Chinese Americans’ diabetes dietary self-management practices are poorer than those of Black and Hispanic Americans [8]. For instance, a study found unfavorable levels of diabetes self-management among Chinese American participants with T2D, reporting that only 36.8% followed dietary recommendations [9]. Chinese Americans’ tendency to consume carbohydrate-heavy meals impedes their ability to follow nationally recommended fiber intake. For example, a review of Chinese immigrants’ dietary behaviors in the United States found that fiber consumption ranged from 12 to 14 grams per day, which is less than the minimum recommended 25 grams per day for women and 38 grams per day for men [10]. Challenges to practicing diabetes dietary management are exacerbated by several cultural factors specific to Chinese Americans, for example, lack of culturally adapted dietary programs [5], disruptions in meaningful cultural meal practices that are normally carbohydrate-rich (such as rice), and eating with others in a communal manner coming from a collectivist culture [5].

Similar challenges in diabetes dietary self-management have been observed in other immigrant and ethnic minority populations. For example, Iqbal [11] conducted a scoping review of dietary behaviors among British South Asians with T2D and identified a range of cultural influences that complicate dietary self-management. These included strong cultural and religious norms surrounding food preparation and consumption, such as expectations to eat traditional high-carbohydrate meals (eg, white rice, roti, and sweets), reluctance to reject food offerings due to hospitality norms, and communal eating practices. Many participants expressed skepticism toward Western dietary advice and emphasized the importance of culturally tailored counseling. The study highlighted the need for diabetes education that respects cultural identity, incorporates traditional foods in a health-conscious way, and involves family members in intervention efforts. Many of these barriers have also been reported among Mexican Americans, who often face challenges reducing consumption of culturally important foods, such as tortillas, rice, and sweetened beverages, despite understanding dietary risks [12]. In African American communities, dietary self-management may be hindered by systemic issues, including food insecurity, neighborhood-level food access, and culturally embedded food preferences and preparation styles [13]. Collectively, these findings reveal that culturally responsive and community-informed strategies are essential to support dietary self-management in diverse populations with T2D.

Wearable devices can monitor a wide variety of physiological outcomes, including blood glucose, blood pressure, blood lipid levels, weight, waist circumference, and BMI. Among such technologies, studies have shown the continuous glucose monitor (CGM) to be an effective tool in capturing glucose patterns and positively influencing users’ dietary choices. CGM technology is already well-established in the management of type 1 diabetes (T1D), where it has been shown to improve glycemic control, reduce hypoglycemic events, and enhance quality of life. A study has demonstrated improvements in time-in-range and reduced glucose variability when CGMs are paired with insulin delivery systems [14]. Similarly, the development of hybrid closed-loop systems, automated insulin delivery devices informed by real-time CGM data, has significantly advanced diabetes care for individuals with T1D [15]. These innovations have informed the growing use of CGM in T2D management, particularly among patients on insulin or with poor glycemic control. As such, CGM use has evolved from a primarily clinical monitoring tool to a behavioral intervention capable of enhancing patient engagement in both T1D and T2D contexts. For instance, Zahedani et al [16] integrated CGM data within a smartphone app that allowed users to log their food intake, physical activity, and body weight. The app provided daily insights, including glucose pattern tracking, macronutrient breakdown, glycemic index, glycemic load, and activity measures [16]. They found that CGM data integration within a smartphone app enhanced the metabolic health of patients with T2D by significantly improving hyperglycemia, glucose variability, and hypoglycemia. Moreover, users’ body weight decreased, and users developed healthier eating habits, with reduced daily caloric intake and carbohydrate-to-calorie ratio and increased intake of protein, fiber, and healthy fats relative to calories [16].

Other dietary tracking tools have been developed to reduce dietary management challenges. For example, the electronic button (eButton), a wearable imaging device, can automatically record food data during a meal by taking food pictures every 3‐6 seconds, which are then saved on a SIM card [17]. The recorded food picture data are processed to determine food names, volumes/portion size, and nutrient value (eg, carbohydrate grams) [18,19] Studies have established its acceptability and feasibility in real life for dietary assessments; however, the existing device has not been applied to dietary management for individuals with T2D [20,21].

Despite the advances that have been made in terms of using wearable devices in dietary management among patients with T2D, there are a very limited number of studies that consider applying such technology to Chinese American patients with T2D [22]. As Chinese Americans are at notable risk for developing T2D and endure challenges in managing their condition, it is critical to reduce diabetes disparities in this population by understanding how these technologies can be leveraged to improve dietary self-management. Moreover, while CGMs have shown promise in diabetes care, there is limited evidence on their integration with dietary tracking tools (eg, image-based wearable devices like the eButton) among Chinese Americans. Addressing this gap is important to advancing culturally tailored digital health solutions. Thus, this study aimed to explore the barriers and facilitators of using wearable sensors, specifically the eButton and CGM, for dietary self-management for this population. Our research question of interest was: What are the experiences of using wearable sensors (ie, eButton and CGM) for dietary self-management among Chinese Americans with T2D?

## Methods

### Study Design

The study was conducted from January 2022 to October 2023. We conducted a one-group, single-site, prospective cohort study among Chinese Americans. Following instructions on its use, participants were asked to wear a Freestyle Libre Pro CGM for 14 days, wear an eButton to record food images during meal times over 10 days, and record their food intake using a paper diary. On the first day of the study, participants were instructed to wear the eButton on their chest and turn on the camera during meals over the 10-day study period. In addition, our research staff put on the Libre Pro CGM for participants during a 14-day study period. At the end of the 14-day study, research staff removed the CGM from participants, downloaded the CGM and eButton image data, and reviewed the CGM results alongside participants’ food diaries and eButton pictures to help identify potential factors influencing their glucose levels. Following this review, individual interviews were conducted. This protocol enabled a comprehensive assessment of participants’ dietary behaviors and glucose patterns, which informed the subsequent qualitative interviews.

### Participant Eligibility

#### Inclusion Criteria

Participants were eligible to participate in the study if they met the following inclusion criteria: (1) aged ≥18 years old, (2) diagnosed with T2D at least 1 year prior, (3) self-identified as first- or second-generation Chinese immigrants, (4) felt comfortable communicating in English, and (5) had access to a computer and internet connection. While we acknowledge that there is a group of Chinese Americans who are limited in English proficiency, for the limited scope and timeline of this study, we will only focus on those who feel comfortable communicating in English.

#### Exclusion Criteria

Participants were ineligible for participation in the study if they met any of the following criteria: (1) planning frequent travel or vacations or intending to relocate in the next 5 weeks, (2) having serious diabetes-related complications, physical illness, or a mental illness (eg, schizophrenia, bipolar disorder, or substance abuse) that would preclude participation, or (3) having severe cognitive impairments (eg, dementia or intellectual disability).

### Participant Recruitment

We applied convenience sampling to recruit study participants. Eligible participants were recruited using NYU Langone Health electronic health records patient portal (MyChart, Epic Systems Corporation). MyChart recruitment through the NYU DataCore analytics team involves sending a message about the study through the patient portal “MyChart” to patients who meet inclusion criteria. Individuals responded “interested” or “not interested.” Interested individuals were contacted by our research staff for further screening. Qualified individuals who provided consent were invited to the research facilities to learn more about study procedures and conduct research activities. The final sample size was determined based on data saturation; no new themes emerged, which is considered the gold standard of the qualitative method [23,24].

### Qualitative Interviews

At the end of the 14-day study, participants participated in a semistructured individual interview that included open-ended questions to tell us about their experience using eButton and the CGM. The semistructured individual interview is guided by the Healthy Eating guidelines of the Association of Diabetes Care and Education Specialists (ADCES) 7 Self-Care Behaviors [25]. The interviews were conducted in a private room, audio recorded using 2 digital recorders, and lasted approximately 30 minutes each. Participants were informed that they would not be judged on what they had done or said related to the use of the eButton and CGM and that there was no right or wrong answer. Participants were encouraged to talk about what they believed and what they felt when they used the eButton and CGM.

### Relevant Areas Explored in Interviews (Topic Guide)

Data were collected by qualitative interviews based on a topic guide [26]. The topic guide addressed participants’ eButton experiences (eg, what they liked/disliked, what could be improved for future designs), their CGM experiences (eg, what they liked/disliked and opinions on the CGM report), their previous diabetes self-management practices, their previous experiences with measuring food portion sizes and carbohydrate grams, their opinions on eButton and wearing frequency, their opinions on diabetes/nutrition education (and frequency of sessions), and their insight on which populations would benefit from using eButton and CGM together.

The interview topic guide was modified over the course of the study, using an iterative process informed by the content from previous interviews [27]. For example, we incorporated more questions on “Would you like to use other health monitors (eg, Fitbit for tracking physical activities)? Do you think it will increase the burden for you if using multiple health monitors?” after several participants discussed how wearing the CGM and eButton impacted their daily activities.

### Data Analysis

The recorded content was transcribed using Landmark transcription (Landmark Associates Inc) software. Landmark is a specialized human transcription and translation service for qualitative research [28]. This service allows users to transcribe their data through a secure, HIPAA (Health Insurance Portability and Accountability Act)–compliant platform with 99% guaranteed accuracy. Each transcription file is encrypted with a unique key, which is also encrypted by a separate master key that rotates regularly.

Following transcription using Landmark, a research assistant double-checked the interview transcripts to further deidentify any remaining personal identifiable information. Once personalized information was confirmed to be deidentified, a coding book was developed. Two research assistants read the interview transcripts independently, identifying main themes and subthemes. After discussing these results with a third expert in diabetes management, the research assistants created a codebook in an Excel (Microsoft Corp) spreadsheet to develop the coding framework and refine codes where necessary.

Using ATLAS.ti version 23.2.1 software, the 2 trained research assistants thematically analyzed the qualitative transcripts. Each research assistant independently coded the transcripts using the Excel codebook as a guide and then compared their coding results. Furthermore, a set of pregenerated (a priori) codes (eg, eButton pros/cons, CGM pros/cons) was imported into ATLAS software and described to guide the analysis process. Also, new codes (a posteriori) that emerged during analysis (eg, previous diabetes management behaviors) were described. Moreover, each broad topic in the guide was taken as an overarching category (deductive coding), which was then refined based on the analyzed transcripts (inductive coding) [26]. If new codes emerged, all of the documents were rechecked and coded accordingly. Similarities and differences in coding outcomes were discussed between 2 research assistants (and with the third expert in diabetes management using wearable devices) until a consensus was reached. Once codes were analyzed and reviewed in all interview documents, reports were created showing the frequency with which the codes were used to label data segments in the main themes and subthemes. This step assisted in identifying the most common experiences of participants with wearing the eButton and CGM.

In addition, descriptive analysis was conducted for demographic variables using SPSS version 28.0 (IBM Corp). Continuous variables (eg, age and education) were described using mean (SD). Categorical variables (eg, sex and employment) were described using frequency and percentages. After identifying the qualitative findings, we also reported the counts for the number of themes and subthemes.

### Ethical Considerations

The study was approved by the institutional review board at NYU Langone Health (i21-01714). All participants provided written informed consent to participate in this study. Information about study subjects was kept confidential and managed in accordance with the requirements of the HIPAA of 1996. Participants were compensated with a $100 gift card for their participation in the entire study. There were no costs that the participant incurred as a result of participating in the study.

## Results

### Sample Description

We enrolled 12 eligible Chinese Americans with T2D, 1 withdrew from the study, and 11 completed all data collection. The 11 participants who completed the study are all Chinese Americans, with 8 males and 3 females, the age group ranging from 42 to 77 years (Table S1 in Multimedia Appendix 1). The facilitators and barriers to using the eButton and CGM were summarized in Table S2 in Multimedia Appendix 1.

### Experience of Using eButton Device

Participants recounted their experiences of using eButton for the duration of the study period, discussing their perceived advantages and disadvantages of the device, as well as suggestions for improved functionality. Among 11 participants who discussed the eButton advantages, participants perceived that it was very easy to use (n=6) and facilitates mindfulness (n=6), which helps to increase participants’ sense of control over their eating habits and portion sizes. However, major barriers to using eButton included the lack of privacy from the camera (n=4), difficulties in staging the camera (n=5), and the device’s obtrusive appearance (n=4). Participants suggested that the functionality of the eButton device could be improved by including several features, such as having reminders/notifications to turn off the camera (n=2), incorporating language translations (n=1), synching the data to their iPhones (Apple Inc; n=5), and saving pictures of meals to enable record-tracking (n=3).

### Participants’ Perceived Advantages of eButton Ease of Use

More than half of the participants (n=6) in this study noted the very simple, straightforward nature of using the eButton device and were satisfied with the lack of complicated instructions. Much of this ease is due to the device’s ability to collect dietary intake data by automatically taking pictures, leaving the participants with no effort to manually record the information. For instance, one participant describes the benefits of eButton’s effortless utility:

*The fact that [eButton] is not very impactful was probably the best thing. The only thing is that you have to remember to have it… and then put it on, so that was nice.*

[ID 1001]

eButton is easy to use, as it minimally interferes with participants’ daily lives. Users simply place it on their chest, and the device does the rest of the work for them, eliminating any tedious work on the user’s end. Another participant appreciated not having to be overly conscious about tracking information and found it convenient to leave data collection to the eButton:

*eButton is very easy to use for me, and all you do is just make sure you charge the battery before you use it every time you finished the filming.*

[ID 1005]

eButton’s uncomplicated functionality alleviates any inconveniences with using the device in an effective manner. Participants admired this ease of use and noted it as playing a major role in their positive experience with eButton.

### Increased Mindfulness and Sense of Control of Eating Habits

Even though eButton currently only functions as an assessment tool without providing any real-time feedback, participants think it can serve as a reminding tool for eating habits, specifically their meal portion sizes. For example, a participant appreciated how eButton held them accountable with their carbohydrate consumption by gradually becoming more aware of the amount they were eating:

*eButton in a sense actually makes you conscious. So, when I wear eButton it reminds me that “oh, how much is this rice? How much is that cake?” It’s almost like somebody is sitting there reminding you just by wearing it. So maybe I shouldn’t eat that much.*

[ID 1016]

Simply wearing the eButton makes users more conscious about their eating choices and feel responsible for making a healthy change to their dietary behavior. Furthermore, participants found that this increased mindfulness helped them to maintain steady blood glucose levels throughout the day by deliberately controlling their meal portion sizes. If they were presented with the option of consuming an unhealthy food item, they realized that they could exercise willpower and decide to forego eating it or simply have a smaller amount:

*You know right away this meal doesn’t do you any good, and you can stay away from it. And then next time you have it, you either have a smaller portion or just don’t have it at all. And that way, it can help your blood glucose stay stable.*

[ID 1013]

eButton is pivotal in diabetes self-management by holding users accountable for their eating habits, and by doing so, helps them to pay closer attention to the content and size of their meals.

### Participants’ Perceived Disadvantages of eButton

#### Lack of Privacy

Several participants noted the inconvenience of having the eButton device record and take pictures of every object in its periphery. Some participants’ family members were also concerned about being taped, as it made them feel uncomfortable. Thus, participants had to be extra cautious of what was being recorded to respect their own and their family’s privacy. Doing so was particularly challenging because the eButton has a very small camera and dim lighting, so participants often forgot to turn off the recording. One participant describes the burden of this faulty feature:

*... afterwards you have to remind yourself to turn it off. Then you walk around in the house with [eButton] recording everything with other people around. Then it’s a little intrusive. I worry if something is caught on camera.*

[ID 1016]

Five other participants expressed concerns about the eButton’s working status, as they faced problems with knowing whether the device was on or off. This was particularly stressful to some because they were not confident about whether they were recording their meals properly and/or taping something they were not supposed to.

The lack of privacy presents a barrier to participants feeling completely at ease when using eButton in fear of not knowing what is being caught on camera. This barrier may or may not prevent them from wanting to use eButton as often, unless there is a way to remind them to turn off the camera.

#### Difficulties in Staging the Camera

The eButton camera’s small size poses not only an issue, but also the challenge in staging it to take pictures of the meals. The device’s placement on the user’s chest makes it difficult to position it in such a way to capture a high-quality image:

*The placement of eButton, even with the Velcro stickers, was still incredibly difficult because every time I thought I was aiming it at a good angle, when I looked at the images, it turns out it wasn’t a good angle.*

[ID 1017]

Failing to take high-quality pictures may prevent eButton users from keeping an accurate track of the food they are eating, as the bad angles may exclude certain parts of a meal placed in front of them.

#### Bulky and Obtrusive Appearance

Participants’ experiences with eButton were also negatively affected by the device’s bulky physical appearance, as it hindered their ability to interact seamlessly in their day-to-day interactions. One participant criticized the eButton’s placement on their chest that made it socially awkward when speaking with others.

In addition, when asked how long they would feel comfortable wearing the eButton, several participants noted that the obtrusive appearance of the device prevents them from wanting to wear it for extended periods of time.

*It depends, because if it’s something unobtrusive and beautifully designed that you can wear every day that would be nice… but if it’s that big clunky thing, that’s more complicated… if [eButton] is small and you could make it like an accessory, like a brooch.*

[ID 1011]

eButton’s relatively large size may be bothersome and should be designed more discreetly to facilitate participants’ ease and comfort with using the device.

### Participants’ Suggestions for Future eButton Features

#### eButton Reminders for Participants to Turn Off the Device

While the eButton’s functionality has some disadvantages, participants thoughtfully reflected on some of the ways in which future iterations of the device’s features can be improved to address their concerns. First, the eButton’s relatively small, inconspicuous camera made it challenging for participants to remember when to turn off the device, thus causing privacy issues. To combat this inconvenience, participants (n=2) suggested that the eButton should send a notification that reminds the user when to stop recording their meals to prevent them from taping unrelated content. For instance, 1 participant suggested the following:

*Because usually you wear something and then you forget. And you’re doing something else. You watch TV and you talk to people… you have to find a way to turn it off. A reminder. Either a beep or something. An alert so now I realize I should turn it off.*

[ID 1016]

A notification, either in the form of a friendly reminder or noise, would be beneficial to alerting users when to stop recording. Hence, they can be saved from potentially embarrassing themselves and/or wasting the camera’s battery.

#### Incorporate Language Translations

Since all the participants in this study identified as Chinese Americans, most of them felt that their experience using eButton would be improved if it met their bilingual needs with language translations. One participant even described how they would appreciate advanced features, including the ability to incorporate voice activation in different languages:

*Maybe it can also link iPhone translate. It could be multi-language. That would be really nice too. And also, maybe it could do voice activate or voice control. That would be even better.*

[ID 1013]

Having the ability to translate languages and activate voice control techniques would potentially reduce the language barrier that Chinese Americans often endure.

#### Sync Data to Participants’ iPhones

In addition to incorporating advanced language translation features, participants believed that eButton could be made more feasible for users if it could sync the dietary intake data to users’ iPhones and other smartphone devices. In doing so, users can access their data at any time they wish, rather than waiting to schedule and attend an appointment with their doctor to acquire the results. For example, 1 participant explained that syncing the data to their personal devices would be much more convenient for them:

*Linking [eButton] up to an iPhone would be a lot easier and convenient. Because everybody pretty much has an iPhone or smartphone. That way it’d be easier for them to use.*

[ID 1013]

The ability to link up the iPhone to the eButton is helpful to inform users of their dietary intake data in real-time, as many people often have their smartphones with them as they go about their daily lives.

#### Save Pictures of Meals

Finally, participants (n=3) in this study suggested that future iterations of eButton should include a record-tracking feature allowing users to save pictures of their daily meals.

*If [the eButton] were just taking pictures of what I was eating, and when I was eating it, and keeping – so I would be able to see what I had and then what my body’s response in the blood glucose would be [what I’m] interested in.*

[ID 1019]

Having this record-tracking feature would be especially advantageous for users to refer to the content of their meals rather than relying on their memory. Knowing what they ate would enable users to make connections to how their dietary choices influenced their glucose levels. Consequently, users may be more motivated to change their unhealthy eating habits upon understanding the patterns in their diet and its repercussions on blood sugar levels.

### Experience of Using CGM

Participants described their experiences of using CGM for the duration of the study period, discussing their perceived advantages and disadvantages of the device. Among 11 participants who discussed the CGM advantages, some participants perceived that it is more comfortable and easier to use compared to finger sticks (n=5) and increases mindfulness (n=3), which helps to facilitate positive changes to their eating behaviors (n=5). However, barriers to using CGM included its susceptibility to falling off and/or breaking easily (n=1), its tendency to get trapped in clothes (n=1), and the risk of causing skin sensitivity issues (n=1).

#### Participants’ Perceived Advantages of CGM

##### Comfortable and Easy to Use Compared to Finger Sticks

The participants in this study discussed some of their painful experiences with using traditional finger sticks as a means to self-monitor their blood glucose levels. They recounted the difficulty and discomfort in pricking themselves several times per day. Having experienced this invasive procedure consistently, participants were quick to praise CGM’s ability to noninvasively monitor their glucose:

*I think it’s a fantastic device. I wish I could continue using [CGM] because I know that the inconvenience of using the blood glucose meters, you know, having to prick my finger three times a day, ugh, it’s a nuisance. [CGM] has alleviated the nuisance of having to take my blood glucose every day for three times a day. It makes it easier for me to live my life ideally.*

[ID 1017]

The use of CGM saves participants from having to endure the pain and discomfort from continuously pricking such a sensitive area of their body. CGM’s advanced technology allows users to be at ease when monitoring their blood glucose with simply having the device inserted under their skin in a pain-free manner.

##### Increased Mindfulness

Prior to using CGM, participants in the study were not much likely to be aware of the harms from consuming carbohydrate-rich foods, including noodles, rice, and buns. They tended to underestimate the amount of sugar within these foods and were shocked to realize how high their glucose levels spiked as a result of eating them:

*Okay, I tell myself: “Don’t drink any soda. Don’t eat too much sugar.” But it’s not the case like that. You have to more consider the food, what you eat, like carbohydrates and noodles. That, I didn’t know that, you know. I do know they’re a carbohydrate, but I didn’t know that high… when I see the graph, my reaction very surprised me because it was high… from the chart CGM shows me it’s very useful. So I tell myself: “Be careful [with] what you should eat, you should change your lifestyle a little bit in order to live healthy.”*

[ID 1005]

Being able to look at the graph generated by the CGM and interpret the glucose reading provides users with greater insight into how their body responds to the food’s nutrient content:

*I can get to see like every 15 minutes. You don’t get that from a glucose meter. The glucose meter only tells you at that moment, but this is like every 15 minutes. Actually, [CGM] helps me understand a lot more. You probably can pinpoint it to which food spikes [your glucose] the most. Some foods are really good or bad. [CGM] really gives me a much clearer picture of it, so I think it’s great.*

[ID 1013]

Consequently, users are more readily able to identify which specific foods may have contributed to a glucose spike, thus improving their understanding of how they may need to make dietary changes.

##### Facilitate Positive Change for Future Eating Behaviors

The ability for CGM users to recognize which foods may be contributing to increased blood glucose levels may motivate them to facilitate positive changes to their eating behaviors to prevent future glucose spikes. For instance, a participant noted that viewing a lower glucose reading on the CGM graph motivated them to choose lighter foods:

*And then you have the charts, and your mind is like, “Oh, in the future, I can choose this type of food. It makes me lighter.”*

[ID 1001]

### Participants’ Perceived Disadvantages of CGM

#### Prone to Falling Off and/or Breaking

While CGM has several advantages, there are a few notable downsides to using the device because of its fragile build. For example, 1 participant in the study denounced the CGM’s susceptibility to falling off and/or breaking easily. This was the case with 1 participant who accidentally broke the device after going into the ocean:

*You can’t go in the ocean with [CGM] because… I’m pretty sure that’s what broke the first one… like I taped it up and I covered it, but… I’m pretty sure that the salt water or the waves or something just, it can’t handle it.*

[ID 1001]

Users may have to exercise extra caution in handling CGM and might face limitations in terms of specific activities they can engage in (eg, swimming).

#### Tendency to Get Trapped in Clothes

Another barrier to using CGM is the tendency of the device to get trapped in clothes. Users may find it difficult to take on or off their clothing items, especially if they are tight. The conspicuous nature of the device may make the experience of using CGM less comfortable as users become aware of the CGM on their body:

*Sometimes you take off the sweaters, [CGM] got trapped a little bit. You feel there’s something I have to, yeah, take off.*

[ID 1016]

CGM users may have to rely on wearing looser-fitting clothing to allow the CGM sensor to prevent getting caught.

#### Skin Sensitivity Issues

Finally, wearing the CGM may put users at risk of experiencing skin irritation, whether it is a mild rash or a more painful allergic reaction to the adhesive. Skin sensitivity issues are problematic because this could worsen the users’ quality of life and may even prompt the user to discontinue using the CGM, which may impede their diabetes self-management. For example, one participant noted their concern:

*My only concern is my sensitivity to the tape.*

[ID 1006]

Being sensitive to the CGM could possibly deter users from keeping the device on. This problem should be addressed by CGM manufacturing companies to make the adhesives more skin-friendly and avoid dermatological discomfort.

## Discussion

### Principal Findings

This study aimed to explore the experience of using eButton and CGM for improving dietary self-management among Chinese Americans with T2D, a population that has been shown to have greater challenges in dietary management. Participants reported a largely positive experience using the eButton, finding it very easy to use and feeling that it enhanced their mindfulness and sense of control over their eating habits. Participants were also pleased with using CGM, as this device was reported to be more comfortable and easier to use compared to traditional finger sticks, increase mindfulness, and facilitate healthier changes for future eating behaviors. However, the eButton’s lack of privacy, difficulty in staging its camera, and its obtrusive appearance made the experience of dietary self-management less enjoyable. Furthermore, the CGM had several setbacks, including its susceptibility to breaking and/or falling off, its tendency to get trapped in clothes, and its causing some skin sensitivity. Prior studies exploring the experience of CGM use among patients with T2D have reported similar drawbacks, such as skin irritation, rashes, and even bleeding at the attachment site [29].

While there is limited research that explores the experience of using wearable devices for dietary management among Chinese Americans with T2D, existing literature on using such technology among other racial/ethnic minority populations for dietary management demonstrates similar findings. For example, Sabben et al [30] found that some barriers to wearing a CGM among African Americans were related to technical issues (eg, losing signal and malfunctioning), not finding a comfortable and discreet place on the body for the sensor, concerns about infections, and finding the device too complicated to use. Despite these challenges, participants indicated the benefits of CGM, including the convenience of having the longitudinal data available to their health care teams, which helped streamline some of the conversations during doctors’ appointments, as the health care professional could review the data ahead of time [30]. Moreover, Peyyety et al [31] found that the CGM size and visibility are potential barriers to continued usage. Participants reflected on the stigma surrounding T2D and how wearing a CGM could draw unwanted attention to their diagnosis, thus making them uncomfortable and potentially limiting their social interactions with others. However, in terms of feasibility, they also found that participants overwhelmingly favored the convenience of using CGM over finger sticks, similar to what participants in our study had confessed [31]. Similarly, Litchman et al [32] found that a combination of CGM with an online peer support community was feasible, acceptable, and satisfactory for Hispanic adults with T2D. In addition, our study also shows that participants reported a positive experience using the eButton, particularly that it is very easy to use without extra effort for participants to record food intake, as the eButton can automatically record food data by taking food pictures. Although studies have established its acceptability and feasibility in real life for dietary assessments, this is the first study to apply this tool to individuals with T2D [20,21]. This novel application suggests that the eButton may be a feasible and acceptable tool for dietary self-management in this population, particularly when integrated into culturally tailored diabetes care.

Beyond the US context, international research further supports and enriches our findings. Babazadeh et al [33], in a study of Iranian adults with T2D, identified predictors of self-care behaviors and glycemic control, such as self-efficacy and perceived barriers, factors also reflected in our participants’ experiences. Some participants in our study (n=5) described how the ability to recognize which foods caused increases in their blood glucose levels through reviewing CGM report data helped them make positive changes for future eating behaviors to prevent future glucose spikes.

Moreover, a qualitative study by Chowdhury et al [34] in rural Bangladesh identified key barriers and facilitators influencing dietary self-management behaviors among patients with T2D, including gaps between knowledge and implementation, cultural dietary practices, resource limitations, and the role of social and religious support systems. While our participants used wearable devices like CGMs and eButtons to enhance dietary awareness, cultural eating practices—such as sharing carbohydrate-heavy meals with family—sometimes conflicted with the feedback these tools provided. In contrast to our US-based setting, where device usability, appearance, and privacy were key concerns, patients in Bangladesh struggled more with access to healthy foods, structured care, and educational resources. Chowdhury et al [34] emphasized the value of delivering self-management support through trusted, locally embedded health care structures—suggesting that future interventions involving wearable technologies may be more effective when paired with culturally familiar and community-based delivery models in Chinese American populations as well.

### Limitations

The main limitation of our study is the small sample size (n=11) that was enrolled from just a single health care system (ie, NYU Langone), which constrains the transferability of our findings. The limited number of participants may not capture the full range of experiences or perspectives relevant to the topic. Consequently, the themes identified may reflect the specific contexts and individual narratives of our participants, rather than being broadly representative.

Another important limitation is our inclusion of only English-speaking participants. This criterion may have excluded individuals from linguistically diverse backgrounds, who may experience unique barriers or hold different cultural beliefs that influence their engagement with the topic under study. As a result, the findings may not adequately reflect the experiences of non-English-speaking or limited-English-proficiency populations, which is particularly important in multicultural or multilingual settings.

Together, these limitations suggest that while our study provides valuable in-depth insights, caution should be taken when applying these findings to broader or more diverse populations. Future studies with larger, more linguistically and culturally diverse samples are needed to enhance the richness and applicability of the findings across different contexts.

### Strengths

However, our study has notable strengths, including the fact that the collection of dietary intake data was recorded using the eButton, so participant recall bias was not an issue. Moreover, the creation of a comprehensive interview guide that was iterated throughout the process of interviewing participants was helpful to address their experiences, perspectives, and opinions. This approach gave us more flexibility in exploring unexpected insights. In addition, to the best of our knowledge, this is the first study to apply the eButton and CGM to dietary self-management for Chinese Americans with T2D. Our findings suggest that applying eButton in conjunction with CGM may be a promising educational tool to allow individuals with diabetes to understand how their dietary intake impacts their postprandial glucose levels and glucose variability, particularly the consumption of high-carbohydrate food.

### Perspective for Clinical Practice

This study highlights the potential clinical value of integrating visual dietary data from the eButton and CGMs to help dietary management challenges for Chinese Americans with T2D. Although the eButton is not yet widely available or integrated into routine care, this technology offers a compelling glimpse into how future tools might support dietary self-management by allowing patients to see the direct relationship between food intake and glycemic response. In practical terms, the eButton captures timestamped images of all meals and snacks, while the CGM continuously records glucose levels throughout the day. When these 2 data sources are aligned, they provide a visual and numerical timeline of how specific foods influence postprandial glucose. For instance, a patient might review their CGM data and observe a glucose spike after lunch. By examining the corresponding eButton images, the patient, ideally with the support of a diabetes educator or dietitian, can identify which foods or portion sizes may have contributed to the spike. This integrated approach can make dietary self-management more intuitive and personalized, moving beyond generic dietary advice. As a result, patients can build self-efficacy and make dietary decisions more concrete.

However, it is important to recognize that the ability to derive meaningful insights from these devices depends on structured support. Our study reviewed the CGM data at the end of 14-day use alongside the eButton and food diary data to help participants understand how that eating habit impacts glucose levels. Therefore, expecting patients to independently interpret and synthesize complex, multimodal data may lead to confusion, frustration, or disengagement. As such, health care providers must play an active role in facilitating data interpretation, whether through in-clinic discussions, digital dashboards, or annotated feedback reports. Embedding this support into clinical workflows, especially in the context of culturally responsive care, can help ensure that technology enhances rather than complicates the self-management process.

Clinicians should also remain attentive to the practical and psychosocial limitations reported by participants. Concerns about privacy, comfort, stigma, and device visibility are important considerations, particularly among culturally and linguistically diverse populations who may already face barriers to care. Introducing wearable technologies should therefore be approached through shared decision-making, tailored to each individual’s preferences, lifestyle, and social context.

We need to admit that in this pilot study, we only used the Professional (blinded) version CGM to help participants understand the relationship between food intake and glucose changes. Even though it still helps participants realize that high-carbohydrate intake has a significant impact on glucose changes. In addition, while the integration of CGM and eButton data is not yet standard practice, it represents a promising direction for more personalized and responsive dietary support, as suggested by the participants in this study. As digital health tools continue to evolve, their successful adoption will depend not only on technological innovation but also on human-centered implementation in clinical care.

### Future Research

Future research should examine the application of eButton and CGM in dietary self-management among a larger sample size of Chinese Americans with T2D. It would also be beneficial to test the effectiveness of these two devices in racial/ethnic minority populations, particularly among African Americans and Hispanics, due to the high prevalence of T2D in these groups [35].

### Conclusions

In summary, our findings suggest that it is feasible and acceptable for Chinese Americans with T2D to use the eButton and CGM for dietary management, as both of these devices were overwhelmingly well-received by the participants. Participants perceived the eButton and CGM as tools for diabetes self-efficacy since taking pictures of meals heightened their awareness of food portion sizes and observing blood glucose trends improved their understanding of what foods might be contributing to higher glycated hemoglobin levels. However, meaningful use of these devices requires structured clinical support to help patients interpret complex data, highlighting the need for culturally responsive integration within health care settings.

Future studies should add certain features (eg, saving pictures) of eButton, integrate the unblinded version of CGM to provide real-time glucose values, use larger sample sizes, include non-English speaking Chinese patients, and include a longer duration of wearing eButton and CGM to inform effective dietary management for Chinese Americans with T2D. Ultimately, integrating wearable technologies like the eButton and CGM with tailored clinical guidance holds promise for advancing personalized, culturally appropriate dietary self-management strategies in this population.

## Supplementary material

10.2196/73381 Multimedia Appendix 1 Demographic information of participants (N=11) and summary of participants’ facilitators and barriers to using eButton and continuous glucose monitor.

## Abbreviations

ADA: American Diabetes Association
ADCES: Association of Diabetes Care and Education Specialists
CGM: continuous glucose monitor
eButton: electronic button
HIPAA: Health Insurance Portability and Accountability Act
T1D: type 1 diabetes
T2D: type 2 diabetes
