# Supplementary material for: Experience of Using Wearable Devices for Dietary Management for Chinese Americans With Type 2 Diabetes: One-Group Prospective Cohort Study
Source: JMIR Diabetes. 2025 Oct 2;10:e73381. doi: 10.2196/73381 (PMC12490776; doi:10.2196/73381)
Supplement: Multimedia Appendix 1 [file diabetes-v10-e73381-s001.docx]

Table S1. Demographic Information of Participants (N=11)

| Demographics (N=11) | Mean ± SD or n(%) |
| --- | --- |
| Age, years | 55.6 ± 9.5 |
| Education, years | 16.0 ± 2.8 |
| Male | 8 (72.7) |
| Asian (Chinese) | 11 (100.0) |
| Hispanic or Latino | 2 (18.2) |
| Employed | 9 (81.8) |
| Income, $50,000-100,000 | 5 (45.5) |
| ≥$100,000 | 6 (55.5) |
| Married | 8 (72.7) |
| Primary Language (English) | 9 (81.8) |
| Health insurance covered | 11 (100.0) |
| Medicare | 2 (18.2) |
| Medicaid | 1 (9.1) |
| Private | 6 (54.5) |
| Medical conditions (N=11) | Mean ± SD or n(%) |
| Diabetes history, years | 7.4 ± 3.1 |
| BMI, kg/m2 | 27.2± 4.2 |
| Hyperlipidemia | 6 (54.5) |
| Hypertension | 4 (36.4) |
| Coronary heart disease | 2 (18.2) |

SD = standard deviation

BMI = body mass index

Table S2. Summary of Participants’ Facilitators & Barriers to Using eButton & CGM

| **eButton** | | **CGM** | |
| --- | --- | --- | --- |
| **Facilitators**   - Easy to use - Increased mindfulness and sense of control of eating habits | **Barriers**   - Lack of privacy - Difficulties in staging the camera - Bulky and obtrusive appearance | **Facilitators**   - Comfortable and easy to use - Increased mindfulness - Facilitate positive change to eating behaviors | **Barriers**   - Prone to falling off and/or breaking - Tendency to get trapped in clothes - Skin sensitivity issues |

CGM = continuous glucose monitor
